# Understanding working memory as a facilitator of math problem‐solving: Offloading as a potential strategy

**DOI:** 10.1111/bjep.12767

**Published:** 2025-03-21

**Authors:** Josh Medrano, Dana Miller‐Cotto

**Affiliations:** ^1^ University of California Berkeley California USA

**Keywords:** cognitive processes, mathematics, offloading, prior knowledge, problem‐solving, working memory

## Abstract

**Background:**

High working memory capacity is associated with improved mathematical problem‐solving skills. A leading theory about why working memory enhances problem‐solving suggests that capable problem solvers might offload information from their working memory for later use.

**Aims:**

This study examined whether the ability to offload information improved problem‐solving for learners with lower working memory capacity.

**Sample(s):**

The participants consisted of 93 undergraduate students from a mid‐sized university in the United States.

**Methods:**

Participants first took a 10‐problem pre‐test, followed by working memory tasks. They were then split into two groups: one with the option to offload using paper and pencil and one without. As part of a post‐test, they completed 10 math problems.

**Results:**

Results indicated that both the offloading and no‐offloading groups improved over time; however, the effect was greater for the offloading group, according to Hedges' *g*. Although no significant interaction between working memory and condition was found, offloading was useful for specific ranges of working memory skills, according to the Johnson‐Neyman technique. An interaction analysis of pretest and condition also suggests that offloading may be beneficial with increased prior knowledge.

**Conclusions:**

These findings emphasize the importance of considering students' prior knowledge in working memory research. They also demonstrate how external aids influence cognitive processes during problem‐solving.

Decades of research indicate that early proficiency in math is crucial as it strongly influences later academic achievement and career prospects (Jordan et al., 2009; Wrulich et al., 2014). Still, many undergraduates enter college needing to take a remedial math class to fill in gaps in their knowledge (Chen, 2016), and while completion of a series of math classes is required for many STEM majors (Engberg & Wolniak, 2013), this is the case even for general education requirements. Consequently, an urgent need is to identify what predicts early math skills and how best to bolster math skills throughout schooling so that students will be able to successfully execute quantitative skills both in their advanced schooling and in their future endeavours. One predictor of math skills is working memory capacity (Byrnes et al., 2019), a processing and storage resource that enables one to hold information in mind and manipulate information for later use (Baddeley & Hitch, 1974; Baddeley, 2000). Some work indicates that those with low working memory capacity also tend to demonstrate difficulties recalling math facts from a lesson or appropriately applying strategies when solving math problems (Swanson & Beebe‐Frankenberger, 2004). Thus, it may be the case that those who demonstrate low working memory capacity may benefit from instruction in the use of specific strategies to support their working memory capacity. Unfortunately, despite a plethora of evidence suggesting that working memory and math are moderately correlated (Peng et al., 2016), how working memory specifically supports or inhibits math problem‐solving is unclear. Here, we test whether a commonly used external aid, using scrap paper, alleviates some of the working memory load for those who demonstrate low working memory capacity.

## Math difficulty

Arithmetic is one of the earliest skills that contributes to mathematics achievement. These early skills often require simple strategies, such as counting using one's fingers (Geary & Widaman, 1992) or retrieving these answers from memory (Fuson & Kwon, 1992). They also require children to master an understanding of specific math principles. For example, the inversion principle, or the idea that adding and subtracting the same number leaves the original quantity unchanged, appears to emerge as early as pre‐school (e.g. Vilette, 2002). However, young children tend to struggle with the concept of math equivalence, the idea that both sides of an equal sign must be balanced or equal (e.g. McNeil & Jarvin, 2007). These simple arithmetic topics are the foundation for complex arithmetic, which involves solving multi‐digit problems weaving in conceptual and procedural knowledge (e.g. Lee et al., 2017). These early skills remain important in learning later mathematical concepts (e.g. Clements & Sarama, 2014). Indeed, many students struggle with fundamental principles as they continue through schooling, demonstrating challenges into adulthood (Eaves et al., 2019, 2020; Robinson & Dubé, 2013).

To understand math learning better, researchers have posited multi‐level componential frameworks of mathematical cognition, in that general cognitive processes (e.g. working memory) and basic mathematical processes (e.g. single‐digit number comparison) predict proficiency with specific mathematical skills (e.g. simple and complex arithmetic), which then predict overall mathematics achievement (Geary & Hoard, 2005; Gilmore, 2023). The specific processes and the operationalization of achievement differ depending on the question of interest. Here, we attempt to understand how working memory skills facilitate math problem‐solving.

## Working memory and math skills

Working memory is a temporary storage and manipulation system for information (Baddeley, 2000; Unsworth & Engle, 2007). It is pivotal in various cognitive tasks, including academic skill development, language acquisition and managing learning disabilities (Diamond, 2013; Fuchs et al., 2014; Linck et al., 2014). A substantial body of research highlights the role of working memory in shaping math abilities (for reviews, see, Friso‐Van den Bos et al., 2013; Peng et al., 2016; Raghubar et al., 2010). Indeed, meta‐analyses indicate a moderate correlation between working memory and math achievement (*r* = .35; Peng et al., 2016). This relationship suggests that students with stronger working memory tend to perform better in math, potentially due to their ability to manipulate and store numerical information effectively (Blankenship et al., 2015; Geary & Brown, 1991).

Research points to working memory's role in problem‐solving, aiding in managing task goals and filtering distractions (Unsworth & Engle, 2007). Individuals with higher working memory capacity can hold relevant information, discard irrelevant details, and retrieve stored data efficiently (Barnes et al., 2014). Moreover, experts in a domain such as mathematics often leverage their working memory capacity by reorganizing pieces of information and applying appropriate strategies more efficiently than others (Chen & Cowan, 2013; Ericsson & Kintsch, 1995; see Chi, 2006, for review). In this study, we expect that working memory should be related to problem‐solving performance, such that those with higher working memory performance have higher mathematics accuracy.

While the relationship between working memory and mathematics is well documented, further research is needed to elucidate how these cognitive processes mechanistically facilitate or hinder math problem‐solving. By deepening our understanding of these dynamics, educators can develop more effective strategies to improve math education outcomes and enhance students' overall academic success.

## Using external aids in problem‐solving

Some common strategies to help students with problem‐solving struggles include writing things down, drawing diagrams, and using manipulatives (e.g. Carbonneau et al., 2020; Hegarty & Steinhoff, 1997). These strategies scaffold learners' problem‐solving by directly pointing out the relations between the problem, prior knowledge and visual representations. By themselves, visual cues, drawing and sketching also enable learning, comprehension, recall, and problem‐solving (Guo et al., 2020; Mayer, 2005; Miller‐Cotto et al., 2022; Van Meter & Garner, 2005). However, most of this work has been in the science and engineering domains, and in most studies, including those in the mathematics domain, problems are presented as word problems, with instructions or cues that would facilitate problem‐solving. In the current study, we examine the role of using an external aid, scrap paper, in mathematics, with problems that are mostly symbolic (i.e. numbers or symbols that have mathematical meaning).

The aforementioned strategies might support cognitive processes involved during problem‐solving, particularly working memory. That is, scrap paper, diagrams and manipulatives may serve as external memory aids when individuals need to maintain information at one stage in a multi‐step problem. In an experiment, Hegarty and Steinhoff (1997) tested undergraduates' spatial working memory and assigned them to either a Notes or No Notes group. Both groups completed a booklet of 40 items that tested participants' mechanical reasoning, but those in the Notes group were encouraged to write on the booklet (“… Feel free to make notes that are useful to you”), and those in the other group were encouraged not to (“it must be kept clean for other students”). Results showed that there was an interaction between spatial working memory ability and notetaking: Those with lower spatial ability who were given the opportunity to make notes were more accurate than those with lower spatial ability who were not given the opportunity. In the current study, we examined whether being given the opportunity to write out answers/steps to a problem is associated with performance and whether the effectiveness of this strategy differs between levels of working memory capacities. We now turn to the theoretical underpinnings for this perspective.

## Theoretical perspectives

One working memory theory suggests that when the load surpasses its capacity, excess items are transferred to long‐term memory for later retrieval, supported by research linking working memory to long‐term memory (Unsworth, 2016). Higher performance on working memory tasks may indicate more precise and accurate representations in long‐term memory (i.e. semantic and episodic representations), leading to successful retrieval (Jones & Macken, 2015). Additionally, working memory functions as an activated form of long‐term memory, implying their interdependence rather than distinct processes (Cowan, 2019). However, this view assumes pre‐existing information in long‐term memory, which does not directly explain how working memory relates to math skills (Norris, 2017, 2019). Norris (2017, 2019) questioned whether long‐term memory can maintain and manipulate repeating representations, like sequences of numbers (e.g. 1–3–5–3), necessary for tasks like arithmetic. Cowan (2022) argued that retaining information in long‐term memory may involve strategies such as chunking (assembling bits of information into meaningful configurations), verbal recoding (by assigning labels to objects/stimuli) and holistic strategies (creating a meaningful story from pieces of information), all of which occur in working memory but interact with information in long‐term memory (Gonthier, 2021). However, research by Cowan et al. (2015) also suggests that domain‐specific knowledge, like vocabulary or symbolic representations, impacts working memory differently than non‐domain‐specific knowledge, such as unfamiliar symbols, such that there may be other processes or other skills (e.g. strategies) involved that may be common to various tasks irrespective of their content. Here, we control for prior knowledge to account for information in long‐term memory that may be activated during problem‐solving.

The cognitive load theory (Sweller, 2011) posits that task performance is negatively related to the task's working memory demands. Thus, a problem solver may use strategies, such as offloading, to succeed in the task. Offloading may represent a specific variation of the transient information effect (Leahy & Sweller, 2011, 2016; Wong et al., 2012). This effect highlights differences in working memory demands when individuals listen to versus read the same material. While auditory information is transient and quickly fades, written text remains accessible as permanent information. A comparable distinction exists between dynamic content, such as videos or animations, and static visuals, such as pictures or diagrams. Transient information generally imposes a greater cognitive load on working memory compared with permanent information. Offloading involves learners or problem solvers converting transient information into a more permanent form, effectively functioning as a variation of the *transient information effect*.

One perspective linked to the aforementioned models suggests that working memory may be correlated with mathematics skills because those with high working memory capacity may *offload* information to their long‐term memory as they solve problems (Miller‐Cotto & Byrnes, 2020). This perspective, derived from research on the nature of working memory, proposes that working memory tasks not only measure the ability to manipulate, attend to, and store information that is temporarily held in mind; these tasks also provide an index of the ability to retrieve information from long‐term memory (Unsworth, 2010; Unsworth et al., 2012). This theory assumes that when the number of items that must be maintained in working memory exceeds some level, potentially due to insufficient prior knowledge in a domain area, the excess items are offloaded to long‐term memory for later retrieval. This offloading may occur through the strategies mentioned above (chunking, verbal recoding and holistic strategies); that is, the information that is offloaded into long‐term memory through the current task has been passed into working memory and is integrated into information that is already in long‐term memory. For students who demonstrate low working memory capacity, they may be unable to offload this information to long‐term memory, requiring additional learning aids. Here, we test this theory using scrap paper, a commonly used learning aid.

## The current study

This study examines whether the mechanistic role of working memory as it relates to math is due to working memory serving as an offloading strategy. The study's approach involves leveraging strategies from cognitive science and its applications to education. First, we use offloading strategies to limit overloading one's cognitive resources when solving arithmetic problems in an experimental design. Prior research suggests that working memory may serve as an offloading source for individuals with higher working memory performance, and those who present working memory challenges may require additional support (Miller‐Cotto & Byrnes, 2020). Here, we test the offloading perspective when solving arithmetic problems.

Our first research question was whether being given the opportunity to offload affects problem‐solving. We predict that offloading should have a positive effect: if intermediate steps are written down and solved accurately, they are likely to be more accurate on the subsequent and final steps, which should lead to higher accuracy across problems (Chen & Cowan, 2013; Ericsson & Kintsch, 1995).

Second, we asked whether prior knowledge and working memory predict performance. We predicted that prior knowledge and working memory should account for a significant variance in problem‐solving performance, based on previous research (DeStefano & LeFevre, 2004; Kalyuga et al., 1998; Peng et al., 2016; Wei et al., 2012). Having baseline proficiency with the problems likely will lead to greater problem‐solving performance. Such proficiency likely indicates high procedural and/or conceptual knowledge (Hiebert & Lefevre, 1986; Simonsmeier et al., 2022). Participants with better working memory are also likely to have higher math performance, based on previous correlational research (Peng et al., 2016).

Our final research question was whether working memory moderates the effect of offloading. We predicted that working memory would moderate the effect of offloading. Specifically, we hypothesized that among those demonstrating lower working memory, those who were given the opportunity to offload would do better than those who were not (Hegarty & Steinhoff, 1997).

## METHOD

### Participants

Participants were 93 undergraduates, recruited from a mid‐sized university in the Midwest (mean age = 19.83, SD = 1.83; 74% female; 39 freshmen, 28 sophomores, 12 juniors, and 14 seniors; 33 psychology majors) through their psychology department. Informed consent was obtained from all participants. Most participants in the study spoke additional languages other than English (*n* = 72; 77%); about a half of participants' mothers completed at least a Bachelor's degree (*n* = 50; 53%), and a third completed high school/some high school/lower (*n* = 34 or 37%). Power analysis was not conducted; however, a sensitivity power analysis showed that our total sample size would be enough to obtain at least a medium effect size (*d* > .5) and that a total sample size of at least 50 is enough to obtain at least 50% power, given an alpha of .05.

### Measures

#### Working memory

To assess participants' working memory, we used two content‐embedded working memory tasks: ABCD and Numeral Strings (Was & Woltz, 2007). These tasks were created to account only for the task‐relevant (as opposed to task‐irrelevant, in complex span tasks) information involved during working memory tasks. On the one hand, a complex span task might involve remembering letters while calculating arithmetic problems, with only the letters recalled at the end (operation span or OSPAN; Kane et al., 2004). On the other hand, a content‐embedded working memory task might only process and manipulate one type of stimuli (e.g. letter sets). Although content‐embedded tasks are still strongly correlated with complex span tasks, which are more commonly found in the literature (*r =* .75; Zamary et al., 2019), content‐embedded tasks might correlate strongly more with higher‐level cognitive tasks such as reading comprehension and inductive reasoning tasks where participants have to maintain task‐relevant information (e.g. Was et al., 2011; Zamary et al., 2019).

Both tasks were presented on a computer. In the ABCD task, participants were given three statements about the order of the letters A, B, C, and D: one statement referred to the order of A and B (e.g. *A comes before B*), another to the order of C and D (e.g. D does not follow C), and the last one referred to the order of AB relative to CD (e.g. *Set 1 comes after Set 2*). The order of the statements varied between trials. After the statements had been presented, participants selected a response out of eight letter sets, according to their interpretation (e.g. CDAB). There were 24 letter sets overall. Participants' accuracy and RTs were recorded. Reliability estimates were high based on accuracy and RTs (McDonald's ω = .94 and .83, respectively).

In the Numeral Strings task, participants were presented with a string of numbers and were asked two questions, presented one at a time, about the order and the magnitude of the strings. For example, when presented with 9 2 4 8 3 5, they might be asked, *What number comes after 8? What is the sum of the last two numbers?* There were 12 strings overall, each with two subsequent questions. Participants entered their answers, which were all numeric, on the keyboard. Participants' accuracy and RTs were recorded. Reliability estimates were high based on accuracy and RTs (McDonald's ω = .83 and .90, respectively).

For each task, a score was calculated to account for speed and accuracy, as the number of correct responses divided by the total response times for all trials (Was & Woltz, 2007). Whereas working memory is typically indexed by accuracy alone, or sometimes by mean RT for correctly answered items, the adjusted speed index covers more individual differences in both accuracy and RT (Was & Woltz, 2007). Accuracy is still highly correlated with adjusted speed, both in the ABCD (*r* = .94, *p* < .001) and the Numeral Strings tasks (*r* = .78, *p* < .001).

#### Math performance

Participants were provided with a 10‐item math assessment of fraction arithmetic to assess prior knowledge (pretest) and a similar assessment for the offloading manipulation (post‐test). For each assessment, problems were presented on paper on one side. A scrap paper was provided for all participants during the pretest and post‐test for the offloading condition. Items were scored based on accuracy (1 point for each correct item). This task is designed to be solved within 10 min without the mental offloading option (i.e. write things down, draw, take notes). Both pre‐and post‐test had adequate reliability (McDonald's ω = .79 and .76, respectively).

### Procedure

Upon recruitment, participants were given a pretest and then randomly assigned to an offloading (*n* = 47; 77% female) or no‐offloading (*n* = 46; 72% female) condition. Participants were tested individually in a laboratory room. Participants completed the pretest on paper first, followed by the working memory tasks on the computer and the post‐test on paper. At the end of the study, participants were given research credit for their participation.

Participants were first assessed on their baseline math knowledge using a pre‐test, and their working memory using the numeric and non‐numeric working memory tasks. Upon completing these tasks, participants were assigned to one of the two conditions: option to offload versus no option. In the option to offload condition, participants were told, “For this task, you will be asked to solve each of the problems listed below. Please use the sheet of paper and pencil to help you. When you have your final answer, please indicate it on the answer sheet here”. Participants were encouraged to use paper to solve the problems. In the no option, participants were told “For this task, you're going to be asked to solve each of the problems listed below. Please do not write down anything. Simply solve the problems in your head. When you have your final answer, please indicate it on the answer sheet here.”

After completing both the pretest and the working memory tasks, they were administered a post‐test similar to the pretest.

### Data analytic plan

We conducted a regression using a covariance adjustment model predicting post‐test scores when controlling for pretest performance (i.e. prior knowledge). Our prediction model included the offloading condition as a dummy variable (i.e. 1 = offloading, 0 = no offloading) and working memory by condition interactions. First, we conducted a time × treatment interaction to establish pre‐ and post‐test growth. Next, we conducted a working memory × condition interaction, predicting post‐test scores by controlling for the pretest and condition as a dummy variable. We used the Johnson–Neyman technique to explicate interactions and determine regions of significance for our moderator variables, a technique which reveals the range of values of a moderator in which the association between the predictor and outcome was significant, based on an alpha level of .05 (Bauer & Curran, 2005; Hayes & Matthes, 2009). We tested this using the PROCESS package in RStudio. Finally, to probe interactions further, we reported estimated marginal means, which are the *predicted* values of the outcomes at certain levels of the interacting variables, using the ‘emmeans’ package in R.

The full data set, script, and code are available on OSF (Medrano & Miller‐Cotto, 2025).

## RESULTS

Below, our findings are organized by our research questions. Further, we used Hedges' *g*, Cohen's U3, and an improvement index to quantify the change from pre to post‐test. Hedges' *g* provides a better estimate for small sample sizes (Cumming, 2012) and is calculated by dividing the difference between the pre‐ and post‐test mean differences by the pooled variance (U.S. Department of Education, Institute of Education Sciences, 2014). Cohen's (1988) standards of small (.2), medium (.5), or large (.8; see Lakens, 2013) can be used to interpret Hedges' *g*. An effect size of at least .25 is important in education research even if statistical significance is not achieved, per the WWC Procedures and Standards Handbook.

### Establishing baseline equivalence

Overall group task performance is given in Table 1. Task performance by condition is presented in Table 2. No differences between the offloading and no offloading conditions were found for pretest (*t* = .76, *p* = .45) and post‐test scores (*t* = −.85, *p* = .40). No differences between conditions were found for most indices of the working memory tasks, except for the Items Correct in the ABCD task (10.17 for offloading vs. 13.04 for no offloading), and both Mean and Total RTs in the Numeral Strings task (Mean RT: 6014 vs. 5173 ms; Total RT: 2.47 vs. 2.19 min).

**TABLE 1:** Overall task performance.

	*M*	SD	Min	Max
Pretest accuracy (%)	82	15	20	100
Post‐test accuracy (%)	74	15	45	100
ABCD items correct	11.59	7.05	4	23
Numeral strings items correct	16.72	4.44	0	23
ABCD adjusted speed	9.94	6.75	0	26.88
Numeral strings adjusted speed	7.74	3.16	1.89	19.18

**TABLE 2:** Means and standard deviations for the working memory tasks.

	Offloading, *n* = 47	No offloading, *n* = 46	*t*	*p*
ABCD				
Items correct	10.17 (6.59)	13.04 (7.27)	2.00*	.05
Mean RT (ms)	3185 (1426)	3013 (722)	−.73	.47
Total RT (min)	1.27 (.37)	1.24 (.24)	−.44	.67
Adjusted speed	8.64 (6.11)	11.26 (7.17)	1.89	.06
Numeral strings				
Items correct	16.53 (4.70)	16.91 (4.19)	.41	.68
Mean RT (ms)	6015 (2135)	5173 (975)	−2.45*	.02
Total RT (min)	2.47 (.79)	2.19 (.43)	−2.15*	.04
Adjusted speed	7.40 (3.28)	8.09 (3.03)	1.06	.29

*Note*: Mean RT is the mean RT for correctly answered items. Total RT is the sum of RTs for all items.

* *p* ≤ .05.

### Are there differences in learning when comparing offloading to no offloading?

Our first research question addressed whether learning differences existed by condition. To do this, we conducted linear regression with post‐test scores regressed onto condition, controlling for pretest. There was no significant difference between conditions (β = .27, *p* = .11). However, mean effect sizes of pretest‐to‐post‐test improvement suggest a meaningful improvement for both groups, with a larger effect size for the offloading condition (Table 3).

**TABLE 3:** Means and effect sizes by condition.

	*n*	Pretest	Post‐test	Hedge's *g*	*p*_holm_
		*M* (SD)	*M* (SD)		
Offloading	47	.81 (.14)	.75 (.16)	.73	<.001
No offloading	46	.83 (.16)	.72 (.14)	.40	.003

### What predicts learning?

Our second research question addressed whether working memory predicted learning when controlling for prior knowledge. We conducted a linear regression test, including pretest scores and working memory adjusted speed scores regressed onto post‐test scores (Table 4). All predictors accounted for 38% of the variance in post‐test scores (*R* ^2^ = .38; Adjusted *R* ^2^ = .36; *F*(3, 89) = 18.48, *p* < .001), with only the pretest score being the significant predictor (β = .52, *p* < .001).

**TABLE 4:** Effects of pretest and working memory on post‐test scores.

	*B*	SE *B*	β	*p*
Intercept	.27	.07		<.001
Pretest	.51	.09	.52	<.001
ABCD	.003	.002	.17	.08
Numeral strings	.003	.004	.06	.56

*Note*: *R* ^2^ = .38; Adjusted *R* ^2^ = .36; *F*(3, 89) = 18.48, *p* < .001.

A separate model (Table 5) with a composite adjusted speed index of working memory (*M* = 8.84, SD = 4.32, min = 1.29, max = 19.12)—an average of both tasks—was also conducted, as the working memory tasks were highly correlated (*r* = .45, *p* < .001). Results indicated that when controlling for pretest as a predictor (β = .52, *p* < .001), working memory was also a significant predictor of post‐test scores (β = .20, *p* = .03, Δ*R* ^2^ = .03). The regression coefficients indicate that a standard deviation increase in pretest score and working memory is associated with .52 and .20 standard deviation increases, respectively, in post‐test scores.

**TABLE 5:** Effects of pretest and combined working memory on post‐test scores.

	Model 1				Model 2			
	*B*	SE *B*	β	*p*	*B*	SE *B*	β	*p*
Intercept	.27	.07		<.001	.26	.07		<.001
Pretest	.57	.08	.59	<.001	.50	.09	.52	<.001
Working memory					.01	.003	.20	.03
*R* ^2^	.35				.38			
Δ*R* ^2^					.03			

*Note*: Overall Model: Adjusted *R* ^2^ = .37; *F*(2,90) = 28.01, *p* < .001.

### Does either predictor moderate the effect of offloading?

**FIGURE 1:**
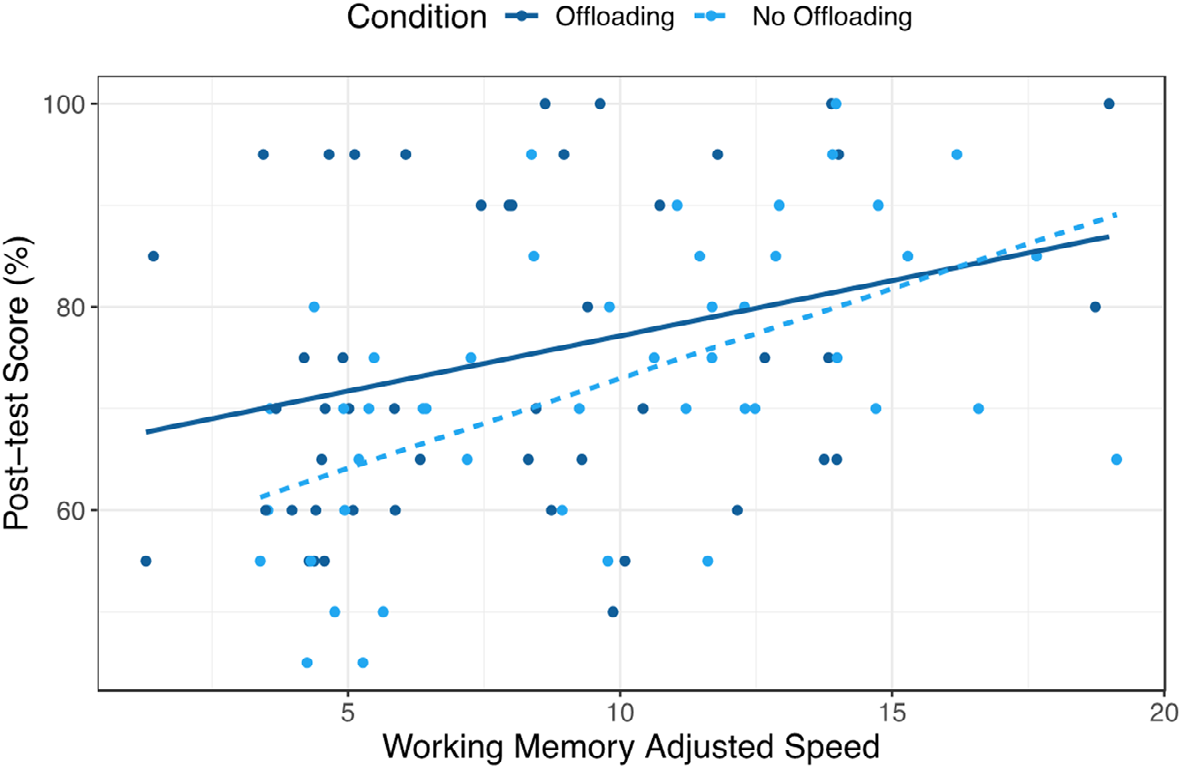
Interaction between working memory and condition.

For the third research question, we were interested in whether working memory moderated the effect of condition on the post‐test score. Working memory was again the average of the two tasks. A linear regression was conducted with post‐test scores regressed on a condition dummy variable for offloading (1 = offloading; 0 = no offloading), pretest, working memory, and a working memory × offloading term (Table 6). We found no significant interaction between condition and working memory (β = −.16, *p* = .35; see Figure 1). Working memory and pretest remained significant predictors when including the interaction term, with working memory and pretest scores associated with increases in post‐test scores (working memory: β = .31, *p* = .01; pretest: β = .52, *p* < .001).

**TABLE 6:** Moderation of working memory on post‐test scores.

	*B*	SE *B*	β	*p*
Intercept	.20	.07	−.19	.007
Offloading	.10	.06	.35	.08
Working memory	.01	.004	.31	.01
Pretest	.50	.08	.52	<.001
Offloading × Working memory	−.01	.01	−.16	.35

*Note*: *R* ^2^ = .42; Adjusted *R* ^2^ = .39; *F*(4,88) = 15.87, *p* < .001.

The interaction was probed further using the Johnson–Neyman technique, which reveals the range of values of a moderator in which the association between the predictor and outcome was significant, based on an alpha level (Bauer & Curran, 2005; Hayes & Matthes, 2009). This is because the conditional effect of X on Y might differ from 0 on specific ranges. The test returned a range in which the conditional effect of offloading (0 = no‐offloading; 1 = offloading) on post‐test scores was significant and positive when values of working memory adjusted speed were between 5.25 and 7.19 problems per minute, *p*s < .05. This finding suggests that offloading was beneficial for individuals who performed worse on the working memory task.

An estimated marginal means analysis was also conducted to examine the predicted values of each condition at different levels of working memory scores. We found no difference in post‐test scores between conditions at a lower level of working memory (adjusted speed = 1 problem/min; 71.9% for the offloading condition vs. 62.5%, *t* = 1.85, *p* = .07). We also found no difference between conditions at a higher level of working memory (adjusted speed = 20 problems/min; 81.9% for the offloading condition vs. 82.8%, *t* = .13, *p* = .90) (See Figure S1). However, using the values from the Johnson–Neyman technique, we found differences between conditions. When the adjusted speed was 5.25 items per minute, the offloading condition performed better than the no‐offloading condition on the post‐test (65.7% vs. 73.5%, *t* = 2.23, *p* = .03); this was also the case when the adjusted speed was 7.19 items per minute (75.2% vs. 69.1%, *t* = 2.31, *p* = .02), supporting the Johnson–Neyman analysis.

**FIGURE 2:**
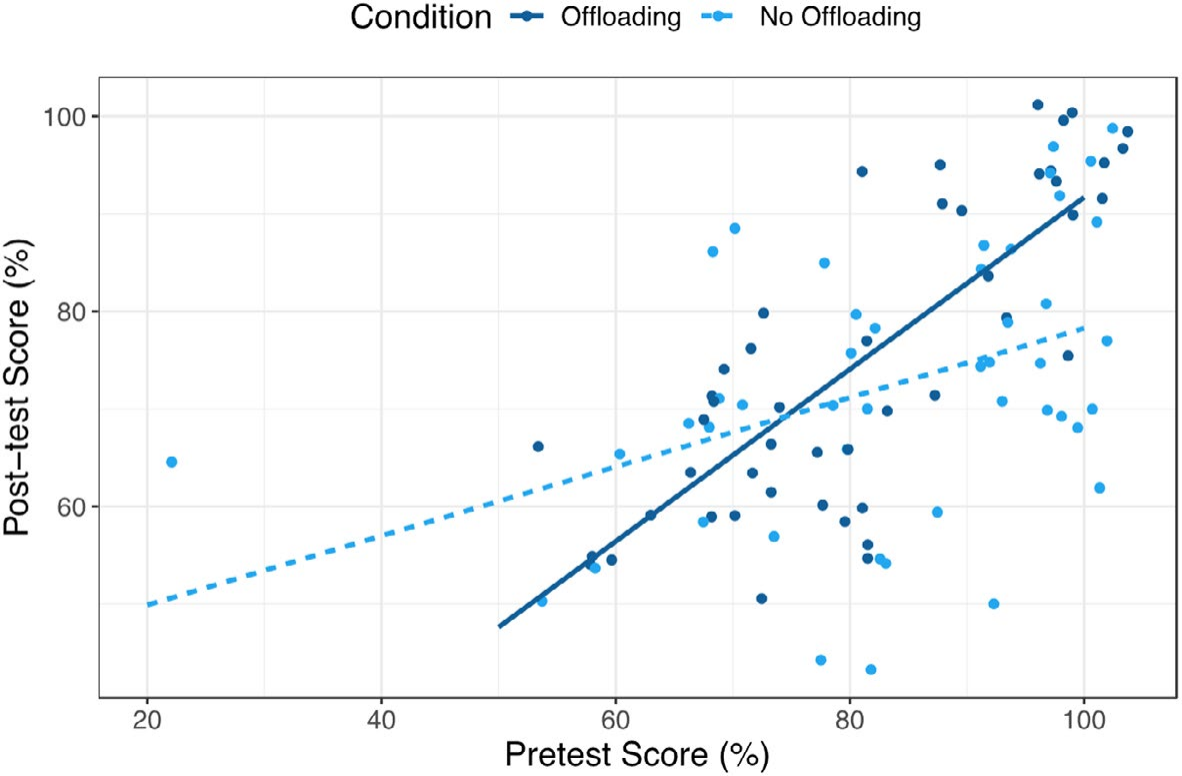
Interaction between pretest and condition.

Due to the magnitude of the effect size for pretest scores on post‐test scores, we examined whether there was a significant interaction of pretest scores by condition (Table 7). Results indicated a significant interaction for pretest scores by condition (β = .54, *p* < .001; see Figure 2). The Johnson–Neyman technique showed that the slope of condition (0 = no‐offloading; 1 = offloading) was significant and negative at pretest scores below 57.61% and significant and positive above 83.16%, *p*s < .05.

**TABLE 7:** Moderation of pretest on post‐test scores.

	*B*	SE *B*	β	*p*
Intercept	.43	.09	−.12	<.001
Offloading condition	−.39	.13	.28	.003
Pretest	.35	.10	.37	<.001
Offloading × Pretest	.53	.16	.54	.001

*Note*: *R* ^2^ = .44; Adjusted *R* ^2^ = .42; *F*(3,89) = 23.26, *p* < .001.

Estimated marginal means were also conducted to examine the predicted values of each condition at different levels of pretest scores (see Figure S2). In contrast to the findings with working memory as a moderator, we found that at the lower end of pretest scores (e.g. 20% accuracy), those in the offloading condition had lower post‐test scores than those in the no‐offloading condition (21% vs. 50%, respectively, *t* = 2.89, *p* = .005); at the higher end of pretest scores (e.g. 100% accuracy), those in the offloading condition had higher post‐test scores than those in the no‐offloading condition (74% vs. 71%, *t* = 3.09, *p* < .001, respectively). Overall, these findings suggest that the offloading group does not benefit more from offloading at lower levels of pretest scores. Instead, students might benefit from offloading with increased prior knowledge.

## DISCUSSION

Although scientists have long established the relationship between working memory and math, the relationship has largely been unexplained, though many theories have emerged (Miller‐Cotto & Byrnes, 2020). The current study examined whether offloading strategies explained the role of working memory in math. The results indicate that students who offloaded also improved to a greater extent than those who did not offload. We did not find differences between students who offloaded and those who did not when also looking at their working memory. Regardless, we found that working memory contributed to problem‐solving, even when accounting for prior knowledge. Finally, we found an interaction between prior knowledge and offloading, suggesting that increased prior knowledge might facilitate the role of offloading in mathematics.

Our work hinges on the theoretical perspective that those with higher working memory allow one to offload information from the task at hand, and allowing students to offload information would facilitate problem‐solving, especially when they have lower working memory capacity and lower prior knowledge (Miller‐Cotto & Byrnes, 2020). The results support prior work suggesting that having the opportunity to offload, such as a scrap paper, facilitates problem‐solving (e.g. Hegarty & Steinhoff, 1997). In particular, students benefited from using an external aid to solve math problems. Students may have been using external aids to accurately recall their prior knowledge, to maintain the solutions to the intermediate steps, or both.

Previous research has also acknowledged the positive role of scaffolds by building on prior knowledge (Rittle‐Johnson & Koedinger, 2009) or providing feedback (Fyfe et al., 2012) in children. One explanation might be that offloading indirectly serves as a feedback mechanism—it helps individuals identify errors and incorrect strategies, which would lead them to correct themselves and apply the correct strategies (Fyfe et al., 2012). Future research should investigate why prior knowledge interacted with offloading. The use of offloading in classrooms can benefit students with enhanced prior knowledge; future research should investigate why this might be.

That prior knowledge and working memory independently predicted performance is also consistent with prior work showing that domain knowledge and cognitive abilities are distinct (Beier & Ackerman, 2005; Hambrick & Engle, 2002; Meinz & Hambrick, 2010; Wittmann & Süß, 1999). It is possible that prior knowledge may also interact with working memory, such that prior knowledge attenuates the relation between working memory and post‐test scores, but previous research has shown this to not be the case (Hambrick & Meinz, 2011; Hambrick & Oswald, 2005). In work with 6‐year‐old UK students (equivalent to first grade in the US), Gilmore et al. (2017) found that working memory, conceptual knowledge and procedural knowledge independently predicted math achievement, and among those with low working memory, there was no additional benefit of having high conceptual or procedural knowledge. These studies, along with the current findings, demonstrate the importance of measuring domain‐general skills when looking at math achievement.

Note that working memory showed a significant contribution only when the tasks were averaged. It is possible that the differences in variability in each task contributed to this effect: performance was more spread out in the ABCD than in the Numeral Strings task (interestingly, using the Items Correct index returned a significant coefficient for the ABCD but not the Numeral Strings). Although the two tasks are correlated, they have been used most often to predict reading comprehension and reasoning abilities in a latent variable context (Was et al., 2011; Was & Woltz, 2007; Zamary et al., 2019). These tasks are content‐embedded, as the output is related to the same information that is being maintained; in comparison, complex span tasks require participants to maintain task‐irrelevant information (e.g. calculating an arithmetic expression while memorizing letters in the OSPAN task; Kane et al., 2004). In Zamary et al. (2019), it was hypothesized and confirmed that content‐embedded tasks (as opposed to complex span tasks) accounted for more variance in an inductive reasoning task because inductive reasoning requires maintaining relevant (as opposed to irrelevant) information. Future research should disentangle the differences between these tasks and their relation to math; just as the relation between working memory and math differs by the math domain (e.g. arithmetic, word problem‐solving, fractions; Peng et al., 2016), it may depend on the type of working memory task (Nozari & Martin, 2024).

Among the strengths of this study was the use of an experimental design to test the role of working memory in math, going beyond correlational research. This gives us confidence that the relation between working memory and problem‐solving is not necessarily related to whether students are offloading information. By manipulating an ecologically or practically relevant educational strategy (offloading), we also address the educational implication of addressing cognitive processes in classroom skills and behaviours. Much research has been done to test the role of working memory itself by manipulating features of mathematical expressions (e.g. Medrano, 2023), but few so far have tested how educational supports affect the relationship between working memory and problem‐solving. Future research might consider a 2 (low/high working memory) × 2 (offloading/no offloading) design or a within‐subjects design to further see whether those with lower working memory benefit from offloading versus no offloading.

Despite these strengths, the study has its limitations. Here, we operationalized offloading as students having the opportunity to solve math problems with scrap paper. However, offloading is not limited to this medium. Subsequent work can investigate other offloading strategies, such as manipulatives, drawing or sketching, fading and worked examples, all of which have been shown to improve learning in math. Additionally, it is also likely that individual differences in offloading may have contributed to problem‐solving performance. The variability of offloading strategies may account for the relation between offloading and problem‐solving; for example, one student may have only offloaded on the second step of a problem.^1^ Finally, there are important factors not accounted for such as math attitude, math anxiety, and metacognitive skills that could account for performance; for example, offloading might be more helpful for those with lower metacognitive skills—instructing those with lower metacognitive skills to offload might lead to greater self‐monitoring, which might then lead to higher accuracy during problem‐solving (Braithwaite & Sprague, 2021). Similarly, offloading might help regulate math anxiety feelings by alleviating working memory resources that might be overloaded due to anxiety itself (Park et al., 2014; Pizzie & Kraemer, 2023). A final limitation is the prior knowledge measure. Here, the pretest and post‐test measures consisted of similar items (fraction arithmetic); it may be interesting to see whether the moderation of prior knowledge also occurs when more foundational conceptual, procedural and factual knowledge items are measured.

### Conclusion

Taken together, the present study found that offloading facilitated problem‐solving performance, and that contrary to our hypothesis, offloading did not interact with working memory. The current study has theoretical implications for the mechanisms underlying the relationship between working memory and mathematics. Most importantly, working memory research should more strongly consider the role of prior knowledge. Here, prior knowledge independently predicted performance, and students may benefit from offloading with enhanced prior knowledge. Regardless, our research pushes forward this area of research by testing a specific hypothesis regarding the relationship between working memory and mathematics.

The study also has practical implications for how to support students in mathematics classrooms. By using external aids, students can more accurately and appropriately apply strategies, solve intermediate steps more correctly, and reflect on the accuracy of their answers. Furthermore, although the effect of offloading did not necessarily differ based on the students' level of working memory, working memory still significantly predicted problem‐solving performance; thus, offloading may also be beneficial to supporting students' working memory during mathematics lessons (Peng & Swanson, 2022).

## AUTHOR CONTRIBUTIONS

**Josh Medrano:** Writing – original draft; writing – review and editing; visualization; methodology; formal analysis; project administration; data curation; investigation. **Dana Miller‐Cotto:** Conceptualization; investigation; writing – review and editing; writing – original draft; methodology; formal analysis; supervision; project administration; resources; funding acquisition; data curation.

## CONFLICT OF INTEREST STATEMENT

The authors declare no conflicts of interest.

## Supporting information


Data S1.

## Data Availability

The data that support the findings of this study are openly available in Open Science Framework at https://osf.io/3ewqd/?view_only=fa36f4f284f04416ab24019af13ba1b1.

## Footnotes

^1^
Due to participants' ID being absent in the students' scrap paper, we were not able to investigate variability in offloading strategies.
