# Supplementary material for: Understanding working memory as a facilitator of math problem‐solving: Offloading as a potential strategy
Source: Br J Educ Psychol. 2025 Mar 21;95(3):871–87. doi: 10.1111/bjep.12767 (PMC12319178; doi:10.1111/bjep.12767)

**Supplementary Material**

**Figure S1**

*Estimated marginal means of posttest scores given certain levels of working memory*


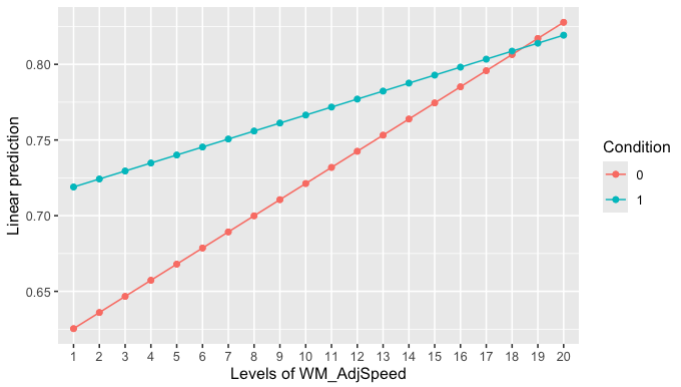


**Figure S2**

*Estimated marginal means of posttest scores given certain levels of pretest scores*


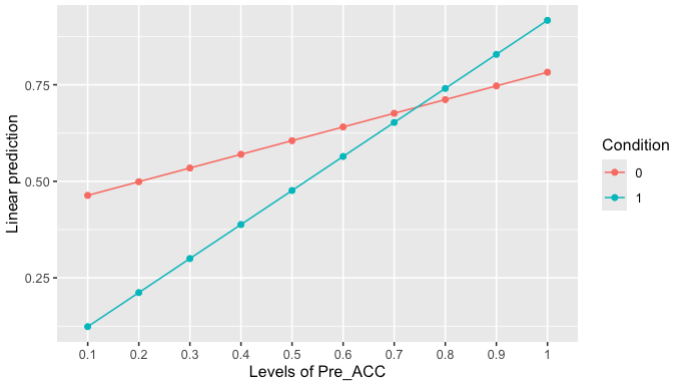

Supplement: Supplementary file 1 — Data S1. [file BJEP-95-871-s001.docx]
